# From the Cochrane Library: Topical Treatments for Cutaneous Warts

**DOI:** 10.2196/33900

**Published:** 2021-12-14

**Authors:** Ani Oganesyan, Torunn Sivesind, Robert Dellavalle

**Affiliations:** 1 Department of Dermatology University of Colorado School of Medicine Aurora, CO United States; 2 Department of Dermatology Rocky Mountain Regional VA Medical Center Aurora, CO United States

**Keywords:** cutaneous wart, plantar wart, topical treatment, papillomavirus infections, evidence-based medicine, wart, treatment, disease management, dermatology

## Research Letter

Below is the body of our research letter. There is no associated abstract due to the nature of our submission.

Cutaneous warts (CWs) are common infections caused by human papillomavirus (HPV) that affect children and young adults. The treatment of CWs aims to relieve associated pain, functional impairment, and psychological discomfort. Lack of data supporting any single curative treatment for the diverse cutaneous manifestations of HPV has created a challenge for healthcare providers in recommending the “most effective” first-line therapy. A 2012 Cochrane Review, “Topical Treatments for Cutaneous Warts,” evaluated treatment outcomes for extra-genital warts in healthy, immunocompetent adults and children, and provides valuable guidance in treatment selection [1].

This analysis [1] compared therapeutic outcomes – namely, cure and decreased incidence of recurrence -- from 85 RCTs (8,815 participants) and reported that salicylic acid (SA) significantly increased the clearance of warts compared to placebo. Data from a meta‐analysis of cryotherapy favored neither intervention nor placebo. Aggressive cryotherapy was more effective than gentle cryotherapy, but with adverse effects such as pain, blistering, and scarring. Metanalysis did not demonstrate a significant difference in effectiveness between cryotherapy and SA, but suggested that combined SA and cryotherapy was more effective than SA alone. Dinitrochlorobenzene was twice as effective as placebo. One study demonstrated local hyperthermia was more effective than placebo in the treatment of plantar warts, but further investigation is necessary to validate these results. Trials of clear duct tape demonstrated no advantage over placebo. Evidence regarding bleomycin was inconsistent. 5-fluorouracil (5-FU) was found to be effective in the treatment of cutaneous warts; however, due to its high side effect profile, its utility is limited to that of refractory cases and elimination of neoplastic lesions. The results of the treatment comparisons are summarized in Table 1.

Notable limitations of this review were that it did not identify RCTs evaluating surgery, formaldehyde, podophyllotoxin, cantharidin, or topical immunotherapy. Furthermore, there was insufficient data to evaluate the use of 80% phenol, 5% imiquimod cream, intralesional antigen, and topical alpha‐lactalbumin‐oleic acid and cantharidin, when not coupled with SA. While there are limited RCTs evaluating the efficacy of intralesional candida antigen, existing studies suggest it’s a viable option in clinical settings and may be particularly helpful in cases nonresponsive to traditional treatment modalities. To provide guidance for the use of these potentially harmful second-line treatments and better characterize efficacy of first-line agents, additional studies with standard end points are necessary.

Recent data supports the successful treatment of small, new-onset warts with SA and cryotherapy, largely due to their safety and simplicity. Regarding recurrent and extensive warts, immunotherapy was shown to be a promising approach to clearing injected warts and those at sites distant to the initial intralesional injection [2-4]. A study [5] comparing immunotherapy to cryotherapy found the former yielded a better therapeutic response with fewer sessions. However, given the novelty of intralesional immunotherapy, further RCTs are needed to compare intralesional immunotherapy options and the associated adverse effects. Lastly, quality of life outcomes associated with each treatment have yet to be determined. Management of warts continues to be a challenge; however, evidence remains strongest for SA and cryotherapy as the safest, most effective initial therapies [1].

**Table 1:** Treatment comparison with respective results, risk ratio, and CI.

Comparison	Result	Risk ratio (95% CI)
SA^a^ vs placebo for all sites	SA was superior	1.56 (1.20-2.03)
SA vs placebo for hand sites	SA was superior	2.67 (1.43-5.01)
SA vs placebo for plantar sites	SA was superior	1.29 (1.07-1.55)
Cryotherapy vs placebo for warts at all sites	Neither intervention nor control was favored	1.45 (0.65-3.23)
Cryotherapy vs placebo for hand sites	Neither intervention nor control was favored	2.63 (0.43-15.94)
Cryotherapy vs placebo for plantar sites	Neither intervention nor control was favored	0.90 (0.26-3.07)
Aggressive cryotherapy vs gentle cryotherapy	Aggressive cryotherapy was superior	1.90 (1.15-3.15)
SA and cryotherapy combined vs SA alone	SA and cryotherapy combined was superior	1.24 (1.07-1.43)
Intralesional bleomycin vs saline injections	No significant difference	1.28 (0.92-1.78)
Dinitrochlorobenzene vs placebo	Dinitrochlorobenzene was superior	2.12 (1.38-3.26)
Clear duct tape vs placebo	Neither intervention nor control was favored	1.43 (0.51-4.05)

^a^SA: salicylic acid.

The notable limitations of this review were that it did not identify RCTs evaluating surgery, formaldehyde, podophyllotoxin, cantharidin, or topical immunotherapy. Furthermore, there was insufficient data to evaluate the use of 80% phenol, 5% imiquimod cream, intralesional antigen, and topical alpha‐lactalbumin‐oleic acid and cantharidin, when not coupled with SA. Although there are limited RCTs evaluating the efficacy of intralesional candida antigen, existing studies suggest it is a viable option in clinical settings and may be particularly helpful in cases nonresponsive to traditional treatment modalities. To provide guidance for the use of these potentially harmful second-line treatments and better characterize the efficacy of first-line agents, additional studies with standard end points are necessary.

Recent data support the successful treatment of small, new-onset warts with SA and cryotherapy, largely due to their safety and simplicity. Regarding recurrent and extensive warts, immunotherapy was shown to be a promising approach to clearing injected warts and those at sites distant to the initial intralesional injection [2-4]. A study [5] comparing immunotherapy to cryotherapy found the former yielded a better therapeutic response with fewer sessions. However, given the novelty of intralesional immunotherapy, further RCTs are needed to compare intralesional immunotherapy options and the associated adverse effects. Lastly, quality of life outcomes associated with each treatment have yet to be determined. Management of warts continues to be a challenge; however, evidence remains strongest for SA and cryotherapy as the safest most effective initial therapies [1].
